# Application of Clinical Aging Indicators for the Assessment of Neurological Health via Screening Among Residents of the Almaty Region

**DOI:** 10.5195/cajgh.2014.168

**Published:** 2014-12-12

**Authors:** Erkin Nurguzhayev, Renat Iliyev, Dmitriy Mitrokhin, Altynay Karimova

**Affiliations:** Department of Neurological Diseases, Asfendiyarov National Medical University, Almaty, Kazakhstan

**Keywords:** aging, cognitive impairment, screening

## Abstract

**Introduction:**

Life expectancy at birth is considered to be a primary indicator of public health success. However, an increase in life expectancy is meaningless if it is not accompanied by an equivalent increase in the number of life years without disability such as physical, cognitive, and psychological abilities. The main consequences of disease leading to neurological dysfunction are directly related to issues such as the inability to walk, talk, learn, live in society, or take care of oneself. The objective of the study was to conduct a medical examination of elderly people as a part of the scientific program “Development of a model (program) of anti-aging to provide active longevity of elderly people of Kazakhstan.”

**Methods:**

As part of a pilot study, we assessed the presence of the following clinical indicators of aging: cognitive impairment (MMSE test), pyramidal symptoms, and ataxia. We conducted medical examination (screening) among 150 elderly persons in Almaty City Polyclinic #8 and 287 elderly persons in Central Regional Clinic of Rayimbek Area, Almaty region aged 45 and above.

**Results:**

The results show that the intensity of changes is directly dependent on the age of the study groups. The cognitive function is the most affected and depends on the age of examinees. The changes are more expressed among residents of Almaty region. The average MMSE score in Almaty was 28.2 (age group of 45–49 years) and 25.8 (age group of 80 and above), and 27.3 and 24.0 respectively in Almaty region. The various symptoms among residents of Almaty tend to stabilize after 65 years, however, the frequency of ataxia continues to grow and increases significantly after 75 years.

**Conclusions:**

Considering that important risk factors of neurological disorders are cerebrovascular diseases of various origins (primarily hypertension, atherosclerosis, and diabetes), an adequate treatment of these diseases will increase a healthy lifespan. Furthermore, it is necessary to conduct additional research for possible methods of reducing existing morbidities so that healthy aging can be achieved.

